# Postpartum modern contraceptive use in northern Ethiopia: prevalence and associated factors

**DOI:** 10.4178/epih.e2017012

**Published:** 2017-03-20

**Authors:** Teklehaymanot Huluf Abraha, Alemayehu Shimeka Teferra, Abebaw Addis Gelagay

**Affiliations:** 1 Department of Public Health, College of Health Sciences, Aksum University, Aksum, Ethiopia; 2 Department of Epidemiology and Biostatistics, College of Medicine and Health Sciences, University of Gondar, Gondar, Ethiopia; 3 Department of Reproductive Health, College of Medicine and Health Sciences, University of Gondar, Gondar, Ethiopia

**Keywords:** Contraception, Postpartum period, Women, Ethiopia

## Abstract

**OBJECTIVES:**

The postpartum period is a critical period for addressing widespread unmet needs in family planning and for reducing the risks of closely spaced pregnancies. However, contraception during the extended postpartum period has been underemphasized in Ethiopia. Therefore, this study aimed to assess postpartum modern contraceptive use among women in northern Ethiopia and to identify factors associated with modern contraceptive use in the postpartum period.

**METHODS:**

A community based cross-sectional study was conducted from March to April, 2015. Data were entered using Epi Info version 7 and then exported into Stata version 12 for analysis. Bivariate and multivariate logistic regression models were fitted to identify the determinants of postpartum modern contraceptive use. Adjusted odds ratios (aORs) with 95% confidence intervals (CIs) were calculated, and p-values <0.05 were considered to indicate statistical significance.

**RESULTS:**

Nearly half (48.0%) of women used modern contraceptives during the extended postpartum period. Postpartum modern contraceptive use was significantly associated with secondary and tertiary education levels (aOR, 4.25; 95% CI, 1.29 to 14.00; aOR, 5.36 ; 95% CI, 1.14 to 25.45, respectively), family planning counseling during prenatal and postnatal care (aOR, 5.72 ; 95% CI, 2.67, 12.28), having postnatal care (aOR, 2.36; 95% CI, 1.15 to 4.87), resuming sexual activity (aOR, 9.53; 95% CI, 3.74 to 24.27), and menses returning after birth (aOR, 6.35; 95% CI, 3.14 to 13.39). In addition, experiencing problems with previous contraceptive use was negatively associated with modern contraceptive use (aOR, 0.34; 95% CI, 0.16 to 0.72).

**CONCLUSIONS:**

Low rate of postpartum modern contraceptive use were found in the study area. Therefore, strengthening family planning counseling during antenatal and postnatal care visits, improving utilization of postnatal care services and improving women’s educational status are crucial steps for to enhance modern contraceptive use among postpartum women.

## INTRODUCTION

Postpartum family planning refers to the prevention of unintended and closely spaced pregnancies during the first 12 monthsafter childbirth [1]. The postpartum period is critical for addressing widespread unmet needs in family planning and for reducing the risks of closely spaced pregnancies [2].

Research has shown prenatal visits [3,4], place of delivery [3,5,6], postnatal visits [4,7], family planning counseling during antenatal care (ANC) and postnatal care (PNC) [8,9], resumption of menses after birth [4,10], to be the key predictors of postpartum modern contraceptive use.

According to the World Health Organization (WHO) technical consultation committee for better maternal and child health outcomes, an interval of at least 2-year following a live birth is recommended before becoming pregnant again [11]. Pregnancies occurring within a year of the mother’s previous birth are riskier for the health of both the mother and the child than those occurring later [12], and children born within one year of a previous birth have a higher risk of mortality than those born after longer intervals [13]. Closely spaced births are also associated with increased chances of chronic undernourishment, stunted growth, and infant mortality [12].

In Ethiopia, evidence has been found that nearly half (47%) of all pregnancies occur within a short birth interval of less than 24 months after the preceding birth [14]. Postpartum women are an important group because may not realize that they are at risk of pregnancy even if they are breastfeeding [15]; therefore, concentrating efforts on increasing postpartum contraceptive use among these women could have a proportionally greater impact than focusing attention on other populations. However, in Ethiopia, contraceptive use in the extended postpartum period is under emphasized by policy-makers [16]. Identifying factors that hinder postpartum contraceptive use is essential for urban communities, because the length of postpartum insusceptibility is declining [17], as a result of urbanization, economic development, and social and cultural changes [18].

Studies of practices involving modern contraception and associated factors during the extended postpartum period are limited in Ethiopia, particularly in the area of this study.

Therefore, this study aimed to address the evidence gap on postpartum modern contraceptive utilization and the factors associated with postpartum modern contraceptive use. This study could help the government, family planning programmers, and other stakeholders in developing strategies to reduce closer birth intervals and to maximize postpartum contraceptive use for the health of both the mothers and children.

## MATERIALS AND METHODS

### Study design and setting

A community based cross-sectional study was conducted in town of Aksum, northern Ethiopia, from March 25, 2015 to April 25, 2015. Aksum is located 1,067 km to the north of Addis Ababa, the capital city of Ethiopia, and 248 km from Mekelle, the capital city of Tigrai Regional State. The current total population of Aksum is 60,706, of whom, 30,960 are women. According to information obtained from the town health office, the town contains four *Kebeles* (the smallest administrative units) with a total of 13,910, households. There are two public health centers, one public referral hospital, one Family Guidance Association of Ethiopia clinic, four private clinics, and nine pharmacies providing maternal and other health services to the population. The source population comprised all reproductive-age women who gave birth in the last 12 months prior to the study period who lived in the town of Aksum.

### Sample size and sampling procedure

The sample size was calculated using the single population proportion formula *n= (Z α/2)^2^ P(1-P)/(d^2^)* [19], considering the 39.2% prevalence of postpartum modern contraceptive use in urban Ethiopia [3], the use of a 95% confidence interval (CI), and a 4% margin of error (*d*) [20]. After adding a 5% buffer for non-response, the total sample size was calculated to be 601.

### Sampling procedure

**Figure 1.**
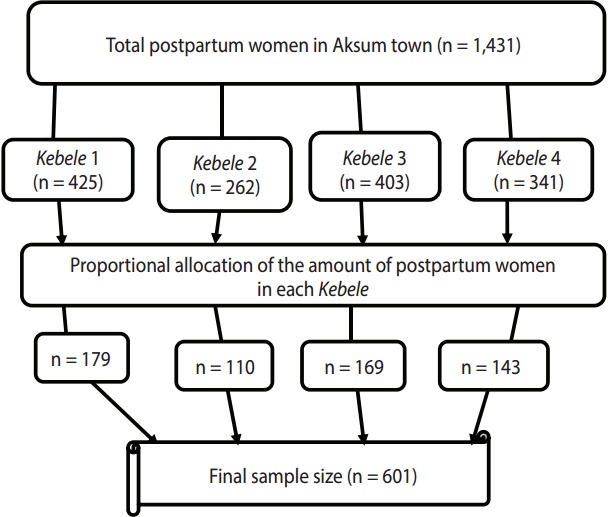
Schematic presentation of the sampling procedure of this study of postpartum modern contraceptive use and associated factors in town of Aksum, northern Ethiopia, June 2015. *Kebele* 1, Hawelti; *Kebele* 2, Kindaya; *Kebele* 3, Hayelom; *Kebele* 4, Maebel.

All four *Kebeles* in Aksum town were selected. The total sample size was allocated by using proportional allocation by size, as applied to the total number of postpartum women in the *Kebeles*. Before the actual data collection, a census was done to identify postpartum women in each *Kebele*. The total number of postpartum women with children under one year of age was 1,431. The study participants were then selected by systematic random sampling techniques. The sampling interval was obtained by dividing the total number of postpartum women in each of *Kebele* by the proportional allocated sample of each *Kebele*. The first postpartum woman was selected by lottery method. Every second postpartum woman was included until the required sample size for each *Kebele* was achieved. If the selected postpartum woman was absent at the time of data collection, the data collectors revisited for two consecutive times, and if the interviewers failed to find the study participant after two visits, the next postpartum woman was included in the study (Figure 1).

### Operational definitions

Extended postpartum period: The 12-month period after a live birth [21].

Postpartum modern contraceptive use: When a postpartum woman reported actively using any modern contraception methods (pill, intrauterine device, injectable, condom [men or women], sterilization [men or women], or implants) during the 12-month following her most recent childbirth.

Knowledge of modern contraception methods: When a woman mentioned at least one modern contraceptive method, she was considered knowledgeable [17,22].

### Data collection instrument and procedures

A structured and pre-tested questionnaire was prepared first in English and translated to the local language (Tigrigna), and translated back to English in order to assess its consistency. Data were collected by eight women diploma holder midwives and one BSc nurse for supervision. Face–to-face interviews were conducted to collect the data. The questionnaire had four parts. The first part included on socio-demographic variables, encompassing age, marital status, occupation, maternal educational status, the partner’s educational level, and monthly family income. The second part assessed reproductive history and maternal health care, including items on ANC utilization, PNC utilization, family planning counseling during ANC and PNC, place of delivery, number of living children, parity, desire for fertility, breastfeeding, husband’s approval, whether the participant had discussed family planning methods with their husband in the last 12 months, current reproductive intentions, birth interval, and the decision to use family planning methods .The third part assessed participants knowledge, and current practices regarding postpartum modern contraceptive use. The fourth part evaluated past experiences with modern contraception services and sexuality related variables, such as family planning counseling by health extension workers in the last 12-month, the experience of any problems with previous modern contraceptive use, whether menstrual periods had returned after birth, and whether the participant had resumed sexual activity since the last birth.

### Data quality control

Data quality was controlled through the provision of training to the data collectors and supervisors about the overall data collection procedures and the techniques of interviewing. A pre-test was done using 5% of the sample questionnaire before the actual data collection in the adjacent town (Wukro Maray Health Center), located 15 km from Aksum, to ensure the clarity of the questionnaire, to check the wording, and to confirm the logical sequence of the questions with a population of postpartum women presumed to have similar socio-demographic characteristics to the population of the study area, and necessary corrections were made based on the pre-test. The collected data were checked for completeness, consistency, accuracy and clarity by the supervisor and the principal investigator on a daily basis.

### Data processing and analysis

All returned questionnaires were checked manually for the completeness and consistency of responses. The collected data were coded and entered in to Epi Info version 7.0 (www.cdc.gov/epiinfo/support/userguide.html) and exported to Stata version 12 (StataCorp., College Station, TX, USA). Data were cleaned and analyzed using Stata version 12 by the principal investigator [23]. For the descriptive analysis, continuous variables were summarized using means, medians, and standard deviations (SDs), while categorical variables were summarized using proportions. Both bivariate and multivariate logistic regressions were used to identify factors associated with postpartum modern contraceptive use. Variables with a p-value< 0.2 in the bivariate analysis were fitted into a multiple logistic regression model to control for confounding effects. Adjusted odds ratios (aORs) with 95% CIs were used to identify factors associated with postpartum modern contraceptive use. The p-values less< 0.05 were considered to indicate statistically significant of the associations with postpartum modern contraceptive use.

### Ethical approval

Before commencement of the study, ethical clearance was obtained from the institutional review board of the Institute of Public Health, College of Medicine and Health Sciences, University of Gondar. A formal letter was obtained from the Aksum town health office administration to obtain permission to conduct the study. Written informed consent was obtained from each study participant to confirm willingness to participate after explaining the objective of the study. Respondents’ names and personal identifiers were not included in the written questionnaires. Education about the importance of postpartum contraceptive use during the extended postpartum period and sources for obtaining contraceptives was provided at the end of the interview for those who did not use postpartum contraceptives.

## RESULTS

### Socio-demographic characteristics of the study participants

Overall, 590 (98.2%) postpartum women participated in to the interview process. The age range of the respondents was 16-49 years. The women’s mean± SD age was 27.4± 5.0 years, and 231 (39.2%) were aged between 25 and 29 years. The majority (92.0%) of the respondents were married. The majority (99.3%) were Tigrai by ethnicity. Of the respondents, 546 (92.5%) were Orthodox Christians. Nearly two-thirds (64.9%) of the respondents were housewives; 236 (40.0%) of women had attended secondary school, and nearly 40.0 % of their partners had attended primary school. The median monthly family income was 1,000 Ethiopian birr per month (interquartile range= 1,400) (Table 1).

### Reproductive health services- related characteristics of the study participants

The mean parity of the study participants was 2.52 (SD, ± 1.46). The mean number of living children was 2.4 per women (SD, ±2.4). Of the study participants, 182 (30.9%) had one child. The median birth interval was 36 months, 86 (14.7%) of the study participants did not intend to have more children in the future. Three-fourths (76.5%) of the women were supported by their husband in using contraceptives. Nearly two-thirds (66.4%) of the women were using modern contraceptives prior to their last child. In addition, 121 (30.9%) had experienced problems using contraceptive methods prior to their last child. More than a third (34.8%) of the women had experienced the returned menses since last birth at the time of the survey. Among all women, 39.4% had resumed sexual intercourse at 6-week of postpartum period and 28.9% at 3-week, and the rest were not resumed (Table 2).

### Maternal health services use -related characteristics of the study participants

At least one ANC visit was reported by (98.1%) of the study participants, and 524 (90.5%) of the study participants had the WHO-recommended 4 or more focused ANC visits for their most recent birth. Of the women, 577 (97.8%) gave birth at health facilities, 258 (43.7%) had PNC follow–up, and 322 (54.6%) had received family planning counseling during their prenatal and postnatal care (Table 2).

### Modern contraceptive use in the postpartum period

**Figure 2.**
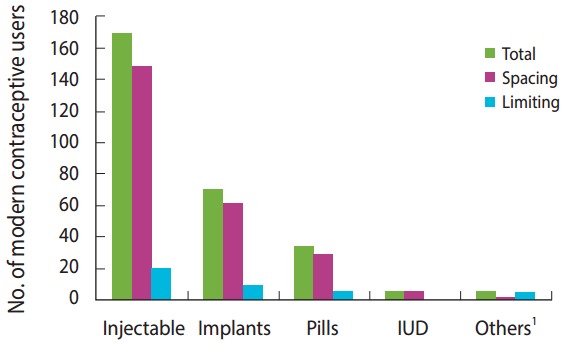
Number of modern contraceptive users by type and purpose among extended postpartum women in town of Aksum, northern Ethiopia, June 2015 (n = 283). IUD, intrauterine device. ^1^Others: women sterilization, men condom.

Modern contraceptive use was reported by 283 women (48%; 95% CI, 43.9 to 52.2%). The most widely used type of modern contraceptive method was injectable contraceptives (59.7%), followed by implants (24.7%) and pills (12.0%). Of the participants, 245 (86.6%) used contraceptive for spacing (Figure 2). Two-thirds (65.5%) of the study participants had started contraceptives before the return of their menses, and 145 (51.2%) of the women had started modern contraceptive by 6-week. Of the women who were using, 254 (89.8%) received their contraception services from government health facilities.

**Figure 3.**
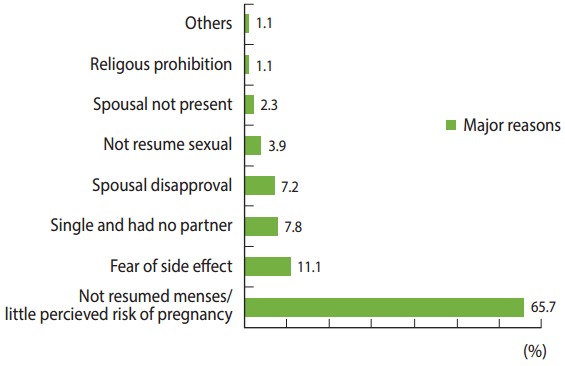
Major reasons for not using modern contraceptives during the extended postpartum period in town Aksum, northern Ethiopia, June 2015 (n = 307). ^1^Others: medical problem, want to have a child soon.

Those who did not report using modern contraceptives, were asked about their future intentions to practice a modern contraceptive method. The majority (84.3%) of such the study participants reported the intention to practice contraceptive use in the future, and 215 (83.3%) planned to use contraceptive for spacing. The postpartum women pointed out various reasons for currently not using contraceptives, such as menses not having resumed, leading to a lower perceived risk of pregnancy (65.7%), and fear of side effects (11.1%) (Figure 3).

### Factors associated with postpartum modern contraceptive use

In the multivariate logistic regression analysis, the following six variables were identified as independently associated with postpartum modern contraceptive use. These were: educational status, family planning counseling during ANC and PNC, receiving PNC, menses returning after birth, resuming sexual activities, and experiencing problems with previous modern contraceptive use. Women who attended secondary school were 4.25 times more likely to report postpartum modern contraceptive use than those with no formal education (aOR, 4.25; 95% CI, 1.29 to 14.00) and those with a tertiary education were 5.36 times more likely to report postpartum modern contraceptive use than those with no formal education (aOR, 5.36; 95% CI, 1.14 to 25.45). Women who received PNC were 2.36 times more likely to use modern contraceptives in the extended postpartum period than compared to women did not (aOR, 2.36; 95% CI, 1.15 to 4.87). Women who were received family planning counseling during ANC and PNC were 5.72 times as likely to use modern contraceptives in the extended postpartum period than those who did not (aOR, 5.72; 95% CI, 2.67 to 12.28). The odds of using modern contraceptive use for women with returned menses was 6.35 times higher compared to those not returned menses (aOR, 6.35; 95% CI, 3.14 to 13.39). The odds of using modern contraceptives in women who had resumed sexual activity were 9.53 higher than in those who had not resumed sexual activity since birth (aOR, 9.53; 95% CI, 3.74 to 24.27). The odds of postpartum modern contraceptive use in women who had experienced problems with previous contraceptive use were 66% lower than in those who did not report such problems (aOR, 0.34; 95% CI, 0.16 to 0.72) (Table 3).

## DISCUSSION

This study was conducted to assess postpartum modern contraceptive use and associated factors among women in the extended postpartum period in the town of Aksum, northern Ethiopia. Nearly half (48.0%) of the women reported using modern contraceptive methods during the extended postpartum period.

Postpartum modern contraceptive use was significantly associated with women’s educational level, family planning counseling during ANC and PNC, utilization of PNC services, menses having returned after birth, resuming sexual activities, and having experienced problem with previous contraceptive use.

This study found that the prevalence of modern contraceptive use among women in the extended postpartum period was 48.0% (95% CI, 43.9 to 52.2). This finding is in line with a studies conducted in town of Gondar (48.4%) [4], in Kenya and Zambia (46%) [24], in Rwanda (50%) [25], and in Mexico (47%) [26].

This finding was higher than that of studies conducted among postpartum women in urban Ethiopia (39.2%), Nepal (40.7%), Bangladesh (37.8%) [3], Uganda (28%) [27], Sri Lankan (41.1%) [28] and India (14%) [5]. This could be attributed to the house-to-house health education strategy through health extension workers deployed in the town by the Ethiopian Federal Ministry of Health.

This study was not in agreement with a study done in Malawi (74.6%) [9]. This difference may be due to the study setting, as the study done in Malawi was institutional-based; it was conducted among postpartum women who came to a health institution for pediatric well care and such subjects could exhibit good health -seeking behavior and have unrepresentative opportunity to obtain health education.

The most popular modern contraceptive methods used by postpartum women were injectable contraceptives (59.7%), implants (24.7%), and pills (12.0%). This indicates that there was a skewed mix of methods in the study population. This can be explained in light of women’s preferences regarding contraceptive methods and health care workers’ attitudes toward contraceptive methods [29]. This is consistent with a study done in Gondar town [4] and a 2011 report by the Ethiopian Demography Health Survey [17].

Almost all postpartum women had a universal knowledge of modern contraceptive methods (95.6%; 95% CI, 93.9 to 97.3%). This suggests that knowledge of contraceptive methods has not yet been translated in to contraceptive practice. This is consistent with the 2011 Ethiopian Demography Health Survey report (97.1%) [17] and a study done in Malawi (94.3%) [9].

This study found that the educational status of postpartum women was significantly associated with modern contraceptive use. This may have been due to the following reasons. First, as the level of educational attainment increases postpartum women are likely to have a better understanding of the available at health facilities and the benefits of fertility regulation. Second, women who have been educated are more likely to visit a health facility and receive counseling or services on family planning, and go on to use modern contraceptives, than who have not been educated. Studies elsewhere have revealed a similar pattern of relationship between educational level and modern contraceptive use [3,5,9,30].

Family planning counseling during ANC and PNC was found to be associated with modern contraceptive use during the extended postpartum period. Women who had received family planning counseling during ANC and PNC had approximately six times higher odds of using modern contraceptives in the extended postpartum period than to their counterparts. This may be because women who are received family planning counseling during ANC and PNC might be highly motivated to use modern contraceptive methods. This result agrees with those of studies conducted in Malawi [9] and North America [8].

Utilization of PNC services was a significant variable influencing the modern contraceptive use in the extended postpartum period. The explanation for this finding is that women who received postnatal care may be likely to obtain family planning counseling and to consider adoption in the postpartum period. This finding is similar to those studies conducted in Gondar [4] and Mexico [7].

Women whose menses returned after birth were 6.35 times more likely to use modern contraceptive than to women with amenorrhea. This is likely because women may be aware of their fertility returning when menses resume. Amenorrheic women would perceive themselves to be less likely to become pregnant, by assuming that amenorrhea would protect against pregnancy irrespective of the postpartum period. Moreover, nearly two-thirds (65.7%) of the women cited the absence of menses as main reason for not using modern contraceptives during the extended postpartum period, and menses returning after birth was found to be a strong stimulating factor affecting the use of modern contraceptives in the postpartum period. This finding is supported by reports from Gondar [4], Nairobi [10], and a Demography Health Survey based-analysis from 17 developing countries [31].

Resuming sexual activity was significantly associated with postpartum modern contraceptive use. Similar findings were reported in studies conducted in Malawi [9], Egypt, Bolivia, and Thailand [32]. The explanation for this may be fact that when postpartum women resume sexual activity, they perceive themselves to be at risk for pregnancy, which motivates them to adopt contraceptive methods. Therefore, resuming sexual activities is strongly linked to the initiation of modern contraceptive use in the postpartum period.

Furthermore, experiencing problems with previous contraceptive use was negatively associated with modern contraceptive use during the extended postpartum period. A study conducted in Malawi supports this finding [9]. This may be due to client dissatisfaction; if clients perceive themselves as having received low-quality services, they tend to discontinue contraceptive use after delivery.

Women stated that their reasons for not using modern contraceptives during the postpartum period were: menses not resuming/lower perceived risk for pregnancy, fear of side effects, single/had no partner, spousal disapproval, and spouse not being present. Similar reasons have been documented in studies conducted in Gondar [4], Malawi [9], and India [5].

In conclusion, low postpartum modern contraceptive use was found in the study area (48.0%). The factors associated with postpartum modern contraceptive use were: maternal educational level (secondary and tertiary education level), receiving family planning counseling during ANC and PNC, having PNC visit, menses returning since birth, resumption of sexual activities at the time of the survey, and having experienced problems with previous contraceptive use. Therefore, strengthening family planning counseling during ANC and PNC visits, improving the PNC services and improvements in women’s educational status are crucial steps for enhance modern contraceptive use among postpartum women.

## Figures and Tables

**Table 1.** Socio-demographic characteristics of the study participants in Aksum, northern Ethiopia, June 2015 (n = 590)

Variables	Frequency	%
Age (yr)		
15-19	19	3.2
20-24	149	25.2
25-29	231	39.2
30-34	126	21.4
≥35	65	11.0
Marital status		
Married	543	92.0
Other^1^	47	8.0
Educational level		
No formal education	80	13.6
Primary school (grade: 1-8)	202	34.2
Secondary school (grade: 9-12)	236	40.0
Tertiary school (grade: > 12)	72	12.2
Partner’s education (n=545)^2^		
No formal education	24	4.4
Primary school (grade: 1-8)	210	38.5
Secondary school (grade: 9-12)	185	34.0
Tertiary school (grade: > 12)	126	23.1
Occupation		
House wife	383	64.9
Government employee	46	7.8
Private employee	103	17.5
Daily laborer	43	7.3
Other^3^	15	2.5
Partner’s occupation (n = 545)^2^		
Government employee	125	22.9
Private employee	257	47.2
Daily laborer	131	24.0
Other^4^	32	5.9
Monthly family income (Ethiopian birr)		
≤600	150	25.4
601-1,000	159	26.9
1,001-2,000	154	26.1
>2,000	127	21.5

1 Single, separated, divorced, or widowed.

2 Among married women.

3 Alcohol /*tella* seller, farmer, or student.

4 Farmer, pension, or guard.

**Table 2.** Reproductive and maternal health service use-related characteristics of the study participants in Aksum, northern Ethiopia, June 2015 (n = 590)

Variables	Frequency	%
Parity		
1-4	536	90.8
≥5	54	9.2
Living children		
1	182	30.9
2-3	281	47.6
≥4	127	21.5
Birth interval (mo)		
<24	105	24.2
24-47	131	31.5
≥48	180	43.3
Reproductive intention		
Want to space	396	67.1
Want to limit	86	14.7
Undecided	99	16.8
Want to have a child	9	1.5
Who decide to use family planning		
Mainly respondents	104	19.1
Mainly the husband	36	6.6
Jointly decision	405	74.3
ANC visits		
1-3	55	9.5
≥4	524	90.5
PNC		
Yes	258	43.7
No	332	56.3
Place of delivery		
Home	13	2.2
Health institution	577	97.8
Postpartum period (wk)		
0-12	186	31.5
13-26	198	33.6
27-38	141	23.9
39-51	65	11.0
Family planning counseling during prenatal and PNC		
Yes	322	54.6
No	268	45.4
Menses returned after birth		
Yes	205	34.8
No	385	65.2
Resumed sexual activities by the time of survey		
Yes	403	68.3
No	187	31.7

ANC, antenatal care; PNC, postnatal care.

**Table 3.** Factors associated with postpartum modern contraceptive use in Aksum, northern Ethiopia, June 2015 (n = 590)

Variables	Postpartum modern contraceptive use		cOR (95% CI)	aOR (95% CI)	p-value^1^
	Yes	No			
Education level					
No formal education	27	53	1.00	1.00	
Primary school (grade: 1-8)	100	102	1.92 (1.12,3.30)	2.87 (0.98, 8.38)	0.05
Secondary school(grade: 9-12)	115	121	1.86 (1.09,3.16)	4.25 (1.29,14.00)^*^	0.02
Tertiary school (grade: > 12)	41	31	2.59 (1.35,5.01)	5.36 (1.14, 25.45)^*^	0.03
Parity					
1-4	269	267	1.00	1.00	
≥5	14	40	0.35 (0.18, 0.65)	0.40 (0.10,1.51)	0.18
Living children					
1	83	99	1.00	1.00	
2-3	157	124	1.50 (1.04, 2.19)	1.40 (0.53, 3.68)	0.49
≥4	43	84	0.61 (0.38, 0.97)	0.81 (0.21, 3.01)	0.76
Currently breastfeed					
Yes	271	304	1.00	1.00	
No	12	3	4.48 (1.25,16.06)	11.01 (0.75, 160)	0.08
ANC visits					
1-3	18	37	1.00	1.00	
≥4	262	262	2.0 (1.14, 3.70)	2.00 (0.59, 6.75)	0.26
FP counseling during ANC and PNC					
Yes	217	105	6.40 (4.40, 9.20)	5.72 (2.67,12.28)^*^	<0.001
No	66	202	1.00	1.00	
PNC					
Yes	173	85	4.10 (2.90, 5.80)	2.36 (1.15, 4.87)^*^	0.02
No	110	222	1.00	1.00	
Postpartum period (wk)					
0-12	43	143	1.00	1.00	
13-26	103	95	3.60 (2.30, 5.60)	1.30 (0.54, 3.27)	0.54
27-38	92	49	6.20 (3.83,10.15)	2.10 (0.77, 5.76)	0.15
39-51	45	20	7.40 (3.99,14.01)	3.80 (0.94,15.27)	0.06
Discussed FP with husband in last 12 months					
Yes	232	209	2.28 (1.45, 3.58)	2.77 (0.67,11.32)	0.16
No	34	70	1.00	1.00	
Husband approval of FP					
Yes	217	200	1.74 (1.16, 2.62)	0.94 (0.26, 3.34)	0.93
No	49	79	1.00	1.00	
Knowledge of postpartum FP					
Yes	280	284	7.55 (2.24, 25.46)	0.37 (0.04,3.21)	0.37
No	3	23	1.00	1.00	
Received FP counseling by health extension worker					
Yes	245	212	2.88 (1.90, 4.39)	0.92 (0.36, 2.35)	0.87
No	38	95	1.00	1.00	
Experienced problems with previous contraceptive use					
Yes	49	72	0.41 (0.26, 0.64)	0.34 (0.16, 0.72)^*^	0.005
No	168	103	1.00	1.00	
Menses returned after birth					
Yes	156	49	6.46 (4.40, 9.50)	6.35 (3.14,13.39)^*^	< 0.001
No	127	258	1.00	1.00	
Resumed sexual intercourse by the time of survey					
Yes	264	139	16.80 (10.00, 28.16)	9.53 (3.74, 24.27)^*^	< 0.001
No	19	168	1.00	1.00	

cOR, cruds odds ratio; aOR, adjusted odds ratio; FP, family planning; ANC, antenatal care; PNC, postnatal care.

1 Hosmer-Lemeshow test.

* p > 0.05.
